# Comparative analysis of diet-associated responses in two rice planthopper species

**DOI:** 10.1186/s12864-020-06976-2

**Published:** 2020-08-17

**Authors:** Hai-Jian Huang, Jia-Rong Cui, Xiao-Yue Hong

**Affiliations:** 1 State Key Laboratory for Managing Biotic and Chemical Threats to the Quality and Safety of Agro-products, Key Laboratory of Biotechnology in Plant Protection of MOA of China and Zhejiang Province, Institute of Plant Virology, Ningbo University, Ningbo, 315211 China; 2 Department of Entomology, Nanjing Agricultural University, Nanjing, 210095 Jiangsu China

**Keywords:** *Nilaparvata lugens*, *Laodelphax striatellus*, Comparative transcriptome, Host adaptation, Diet-associated responses

## Abstract

**Background:**

Host adaptation is the primary determinant of insect diversification. However, knowledge of different host ranges in closely related species remains scarce. The brown planthopper (*Nilaparvata lugens*, BPH) and the small brown planthopper (*Laodelphax striatellus*, SBPH) are the most destructive insect pests within the family Delphacidae. These two species differ in their host range (SBPH can well colonize rice and wheat plants, whereas BPH survives on only rice plants), but the underlying mechanism of this difference remains unknown. High-throughput sequencing provides a powerful approach for analyzing the association between changes in gene expression and the physiological responses of insects. Therefore, gut transcriptomes were performed to elucidate the genes associated with host adaptation in planthoppers. The comparative analysis of planthopper responses to different diets will improve our knowledge of host adaptation regarding herbivorous insects.

**Results:**

In the present study, we analyzed the change in gene expression of SBPHs that were transferred from rice plants to wheat plants over the short term (rSBPH vs tSBPH) or were colonized on wheat plants over the long term (rSBPH vs wSBPH). The results showed that the majority of differentially expressed genes in SBPH showed similar changes in expression for short-term transfer and long-term colonization. Based on a comparative analysis of BPH and SBPH after transfer, the genes associated with sugar transporters and heat-shock proteins showed similar variation. However, most of the genes were differentially regulated between the two species. The detoxification-related genes were upregulated in SBPH after transfer from the rice plants to the wheat plants, but these genes were downregulated in BPH under the same conditions. In contrast, ribosomal-related genes were downregulated in SBPH after transfer, but these genes were upregulated in BPH under the same conditions.

**Conclusions:**

The results of this study provide evidence that host plants played a dominant role in shaping gene expression and that the low fitness of BPH on wheat plants might be determined within 24 h after transfer. This study deepens our understanding of different host ranges for the two planthopper species, which may provide a potential strategy for pest management.

## Background

Most herbivorous insects are restricted to a narrow range of hosts, whereas other insects are adapted to a wider host range [1]. Generally, successful host adaptation involves several essential traits of herbivorous insects, including the abilities to detect the correct plants, acquire nutrients while avoiding intoxication, and overcome plant defenses [2]. Absence of these essential traits have been reported to result in the failure of an insect to feed on specific plants [3–6]. To date, studies of diet-associated responses have mainly focused on a specific insect fed on different diets [7]. The research findings have shown that the genes associated with detoxification, digestion, and transport are significantly influenced by host transfer [7]. However, knowledge of adaptation to disparate hosts in different insect species is scarce, and most studies have primarily focused on differences in detoxification across species in response to plant secondary metabolites [8–10]. Comparative transcriptomic analysis of closely related taxa provides an ideal approach for revealing the distinctions in host adaptation across herbivorous species.

The brown planthopper (*Nilaparvata lugens*, BPH) and the small brown planthopper (*Laodelphax striatellus*, SBPH) are closely related insect pests in the family Delphacidae. Although they both use rice plants as their primary food source, their host ranges differ. BPH is a monophagous insect pest restricted to rice plants, whereas SBPH is an oligophagous insect that can feed on rice, wheat, and other gramineous plants [11]. In a wheat-rice rotation system, SBPH, which is able to overwinter in temperate zones, shifts between rice and wheat plants each year [12]. In contrast, the northern border of the overwintering areas of BPH is at approximately 21–25°N. The migratory BPH is seldom in contact with wheat plants in the locations where BPH overwinters [13]. The driving factors that are potentially implicated in the different host ranges of planthoppers have been investigated for decades. Denno & Roderick suggested that a chemical barrier rather than a physical barrier might prevent BPHs from feeding on non-adaptive plants, as the insects could still settle and insert their stylets into the plant tissues [14]. SBPHs perform better on rice than on wheat plants [15, 16]. Liu et al. found that feeding on different plants significantly influenced the activity of detoxification enzymes in SBPH yet had little impact on BPH [17]. Additionally, the presence of endosymbionts, which provide essential amino acids, was also critical for planthopper colonization [18]. The sap compositions of rice and wheat plants differ, particularly in their amino acid concentrations [19, 20]. These differences in nutrient content influence planthopper performance. Recently, the genomic information for both planthoppers and their host plants (*Oryza sativa* and *Triticum aestivum*) has become available [18, 21, 22], providing an opportunity to study the mechanism of adaptation to different hosts in these insects.

The gut is the main organ where digestion and detoxification occur, and it exerts an important influence on insect feeding on specific plants. Through omics technologies, changes in gut physiology in response to different host plants have been investigated in several herbivorous species, especially the lepidopterans [7]. The expression patterns of gut-associated transcripts, including digestive and detoxifying enzymes, transporters, and peritrophic genes, have been reported to be substantially altered when larvae are exposed to novel diets [9, 23, 24]. For generalist larvae, there is profound transcriptional variation in the gut to overcome the detrimental effects of plant secondary metabolites [24, 25]. For specialist larvae, a specific detoxification system avoids the activation of general stress responses and minimizes the metabolic costs [9, 25], which might restrain a specialist from adapting to a novel phytotoxin [26]. In our previous work, we determined that *CYP4DE1* was critical for SBPH to feed on wheat hosts, with increased mortality observed in ds*CYP4DE1*-treated SBPHs on wheat plants but not on rice plants [15]. *CYP4DE1* expression was upregulated 12 h after transfer to wheat plants. Interestingly, the induction of *CYP4DE1* was specifically detected in the gut but not other tissues [15]. This finding reveals the potential role of the gut in planthoppers feeding on different hosts.

Gene regulation upon an initial exposure (short-term transfer) greatly influences the subsequent adaptation of herbivores to a novel environment [27]. To understand the successful colonization of SBPH on rice and wheat plants, we profiled the gut gene expression patterns of SBPHs colonizing rice plants (rSBPH), colonizing wheat plants (wSBPH), and transferred from rice plants to wheat plants (tSBPH). By comparing the differentially expressed genes (DEGs) of SBPH between short-term transfer (rSBPH vs tSBPH) and long-term colonization (rSBPH vs wSBPH), the rapid response of SBPHs to the change in host plant and the leading role of the host plant in shaping gene expression were documented. To elucidate the differences in host adaptation between BPH and SBPH, the gut transcriptomes of BPHs colonizing rice plants (rBPH) and transferred to wheat plants (tBPH) were also profiled. Based on a comparative analysis of short-term transfer in BPHs (rBPH vs tBPH) and SBPHs (rSBPH vs tSBPH), the potential mechanism of host adaptation in the two planthoppers was examined. This study increases our knowledge of diet-associated responses in herbivorous insects, which might suggest a potential strategy for pest management.

## Results

### Performance of planthoppers on rice and wheat plants

**Fig. 1:**
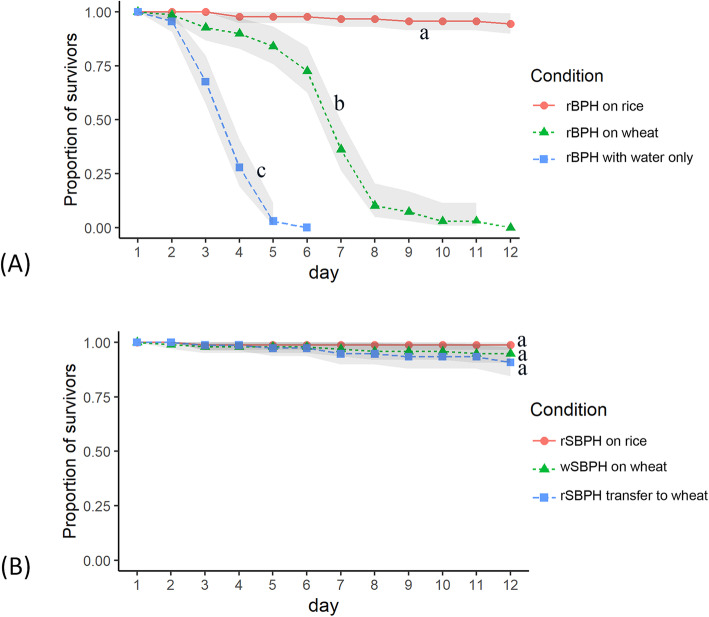
Survival of planthoppers on rice and wheat plants. **a** The survival of rBPHs colonizing rice plants, transferred to wheat plants, and provided with water only. **b** The survival of rSBPH colonizing on rice plants and transferred to wheat plants, and wSBPH colonizing wheat plants. Light shades indicate 95% confidence intervals. Different letters signify significant different survival distributions among each treatment group at *P* < 0.05 according to the log-rank test

Rice plants, but not wheat plants, can be colonized by BPH with only rice-colonizing BPH strains (rBPH) produced under laboratory conditions. In contrast, SBPH can successfully colonize both rice and wheat plants, and two SBPH strains (rSBPH and wSBPH) were maintained in our laboratory for over 30 generations. According to the survival analysis, more than 90% of rBPH survived on rice plants for 12 days, which was significantly higher than that on wheat plants (Fig. 1a). Additionally, rBPH survived longer on wheat plants (LT_50_ = 6.1 days) than those provided with water only (LT_50_ = 3.3 days). These results indicate that BPH could ingest wheat sap and survive on wheat plants for a short time but not for an extended period. For SBPH, both rSBPH and wSBPH successfully survived on rice and wheat plants (Fig. 1b), which is consistent with previous reports [28].

### Overview of RNA sequencing data

To explore the mechanism underlying the different performances of the two planthoppers, rice-colonized planthoppers were transferred to wheat plants for 24 h (short-term transfer) or reared on wheat plants for over 30 generations (long-term colonization). The guts of planthoppers that colonized rice plants (rSBPH and rBPH) or wheat plants (wSBPH) or were transferred from rice plants to wheat plants (tSBPH and tBPH) were then isolated and underwent high-throughput sequencing. A total of 15 libraries (5 treatments and 3 biological replicates for each treatment) were generated, with clean reads exceeding 45 million in each library. The clean reads were mapped to their reference genomes [18, 21]. For BPH, 75–83% of clean reads were mapped to the reference genome. For SBPH, 60–66% of clean reads were mapped to the reference genome. According to the saturation analysis, the number of detected genes decreased as the number of reads increased, and the library capacity reached saturation when the number of sequence reads approached 20.0 million (Fig. S1, Supporting information). Furthermore, principal component analysis (PCA) demonstrated that the expression patterns of wSBPH and tSBPH were closely related, indicating that host plants exerted a nonnegligible influence on gene expression (Fig. S2, Supporting information).

### Analysis of differentially expressed genes (DEGs)

**Fig. 2:**
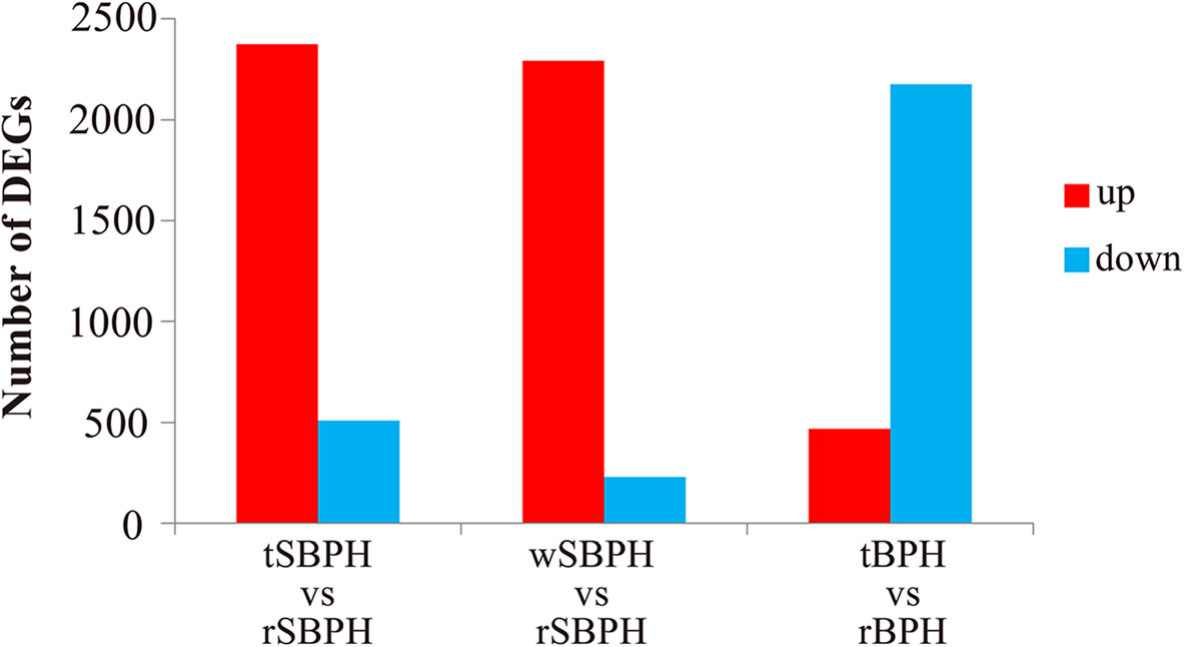
The number of differentially expressed genes in planthoppers that feed on different hosts. The differentially expressed genes were analyzed by comparing tSBPH to rSBPH, wSBPH to rSBPH, and tBPH to rBPH based on a threshold of > 2-fold change and an FDR-adjusted *p*-value of < 0.05

Gene expression changes were analyzed by comparing rice colony planthoppers to transfer planthoppers (tSBPH vs rSBPH and tBPH vs rBPH) and rice colony planthoppers to wheat-colony planthoppers (wSBPH vs rSBPH) using a threshold change of > 2-fold and an FDR-adjusted *p*-value of < 0.05. For rice colony planthoppers transferred to wheat plants, a total of 2877 and 2638 genes were differentially expressed in SBPH and BPH, respectively (Fig. 2). There were 2372 genes upregulated and 505 genes downregulated when rSBPH was transferred to wheat hosts (Table S1). Among these DEGs, genes participating in signal transduction were particularly upregulated. CYP4DE1, which mediates wheat adaptation and ethiprole tolerance [15] in SBPH, was also significantly induced after transfer (Fig. S3, Supporting information). In contrast, 71 genes related to ribosomal proteins and 48 genes related to oxidative phosphorylation were significantly downregulated, indicating decreased protein production and energy metabolism (Fig. S4, Supporting information). In BPH, the number of genes downregulated (2171 genes) exceeded the number of genes upregulated (467 genes) (Table S2). The majority of genes that were significantly downregulated were associated with intestinal mucins, serine proteinases, and sugar transporters. In addition, reduced expression was also found for detoxification-related genes (Fig. S3, Supporting information), which included 9 ABC transporters, 8 P450s, 5 UGTs, and 1 GST. In contrast to SBPH, the majority of ribosomal proteins were upregulated in BPH (Fig. S4, Supporting information). Cuticular proteins, which form the insect cuticle and are involved in insect molting, were dramatically upregulated after BPH was transferred to wheat (Table S2).

In the comparison of rSBPH and wSBPH, a total of 2516 DEGs were identified (Fig. 2). Strikingly, 90.9% of DEGs (2288 genes) showed higher expression in wSBPH than in rSBPH (Table S3), with genes related to peroxisomal biogenesis factor, nucleotide exchange factor, peptide transporter, and CYP6FK1 exhibiting the most dramatic changes. Similar to the patterns of rSBPH transferred to wheat hosts, 37 genes participating in signal transduction were significantly enriched. Among the 228 downregulated genes, the most dramatic changes in zinc metalloproteinase, UGT, and alpha-glucosidase were observed. Other downregulated genes participating in chitin metabolism, carbohydrate derivative metabolism, starch and sucrose metabolism, and oxidative phosphorylation were significantly enriched (Table S3).

### Classification of SBPH genes associated with diet changes

**Fig. 3:**
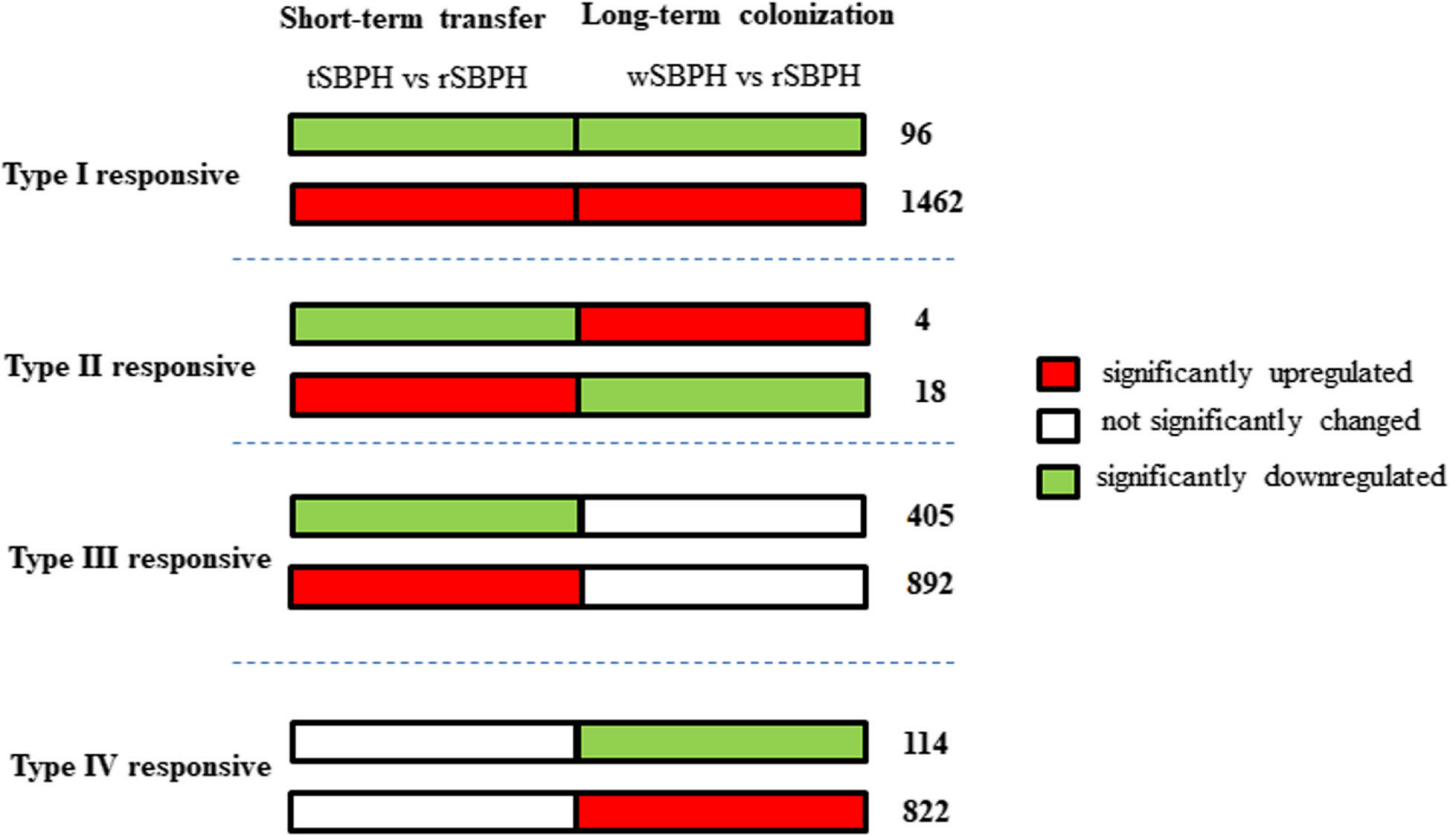
Classification of DEGs in SBPH based on their expression patterns. The expression patterns of identified DEGs in SBPH can be classified into four types: Type I, expression changed in the same direction for short-term transfer and long-term colonization; Type II, expression changed in the opposite direction for short-term transfer and long-term colonization; Type III, expression changed in the short-term transfer, but not in the long-term colonization; Type IV, expression changed in the long-term colonization, but not in the short-term transfer. The number of genes belonging to each type is listed following the heat map. The red and green boxes represent up- and down-regulated genes, respectively, in tSBPH and wSBPH relative to those of rSBPH. The white boxes represent genes that did not change

To elucidate the successful colonization of SBPH on rice and wheat plants, the DEGs of SBPH for short-term transfer (rSBPH vs tSBPH) and long-term colonization (rSBPH vs wSBPH) were analyzed. Based on the gene expression changes in response to different diets, the DEGs of SBPH were classified into four types (Fig. 3): I) genes changed in the same direction for short-term transfer and long-term colonization, II) genes changed in opposite direction for short-term transfer and long-term colonization, III) genes changed in response to short-term transfer but not long-term colonization, and IV) genes changed in response to long-term colonization but not short-term transfer.

There were 1558 genes associated with a type I response (Fig. 3; Table S4). The enrichment analysis showed that genes participating in signal transduction and immune system were significantly overrepresented. Only 22 genes were associated with a type II response (Fig. 3; Table S4). Four genes were downregulated after transfer but were dramatically upregulated during colonization, whereas the other 18 genes showed the reciprocal expression pattern. There were 1297 genes associated with a type III response (Fig. 3; Table S4). The enrichment analysis showed that the ribosome pathway, oxidative phosphorylation pathway, and retrograde endocannabinoid signaling pathway were significantly overrepresented. It was noteworthy that the expression level of ribosome proteins was initially suppressed but recovered when SBPH colonized wheat plants over the long term. A total of 936 genes were associated with a type IV response (Fig. 3; Table S4). However, we failed to find GO terms or KEGG terms that were significantly enriched. Genes such as integrin alpha-PS4-like, integumentary mucin, and proliferation-associated protein showed a higher expression level in wSBPH.

### Comparative genomics in response to host transfer

To comprehend the different diet-associated responses across species, 6139 gene families with only one ortholog in BPH and SBPH were selected and compared. A total of 1995 gene families were differentially expressed in at least one planthopper species after transfer (Table S5), among which 370 genes were responsive to host transfer in both planthoppers. Interestingly, only 22 genes changed (14 genes upregulated and 8 genes downregulated) in the same direction in both species, including heat-shock protein, prophenoloxidase activating factor, MAP kinase-interacting serine/threonine-protein kinase, and small nuclear ribonucleoprotein. Nonetheless, other 348 genes showed different expression patterns between BPH and SBPH. Among these, 293 genes were upregulated in SBPH after transfer, but downregulated in BPH; 55 genes, including 25 ribosomal proteins, were downregulated in SBPH after transfer but were upregulated in BPH.

### qPCR validation

**Fig. 4:**
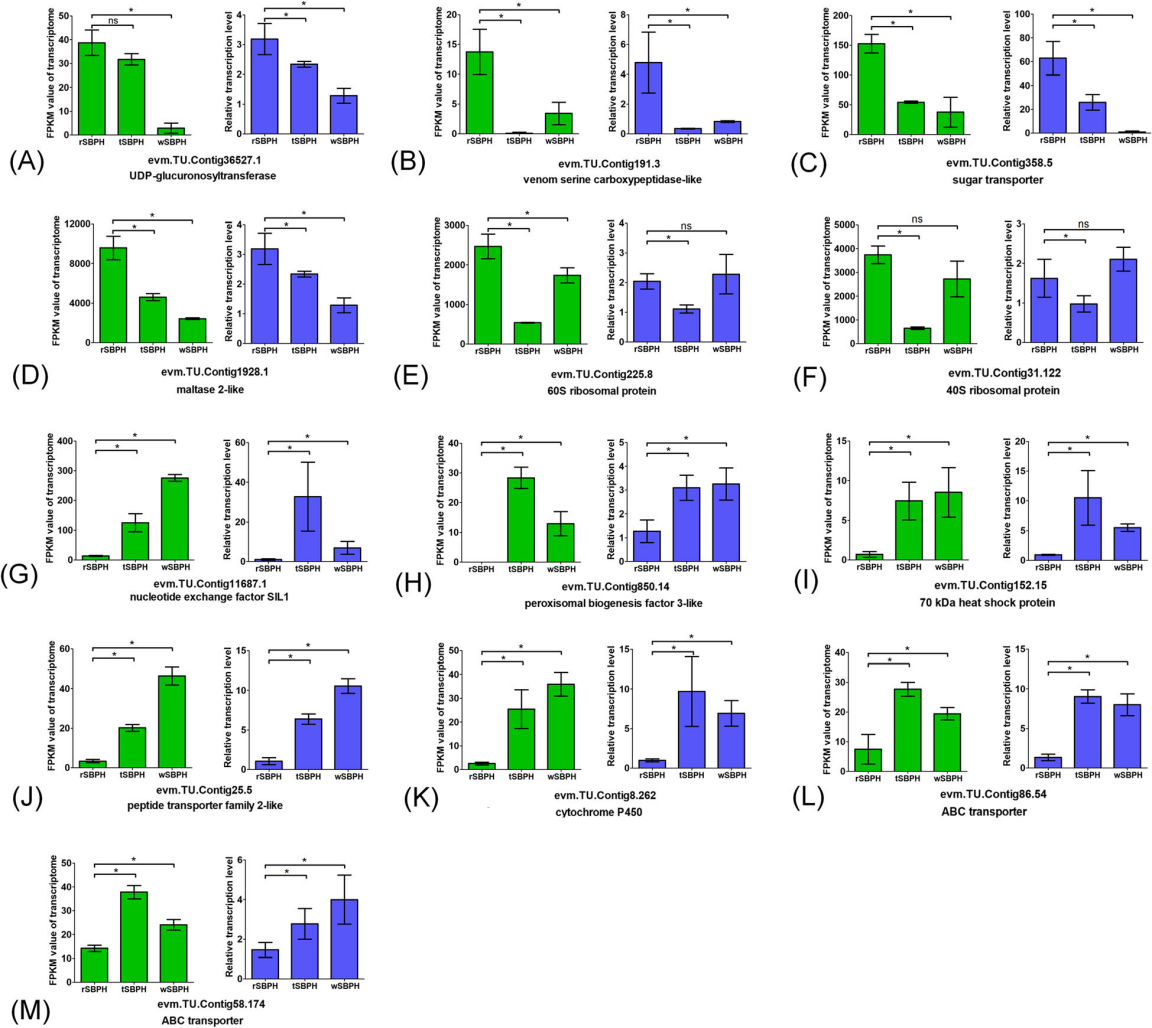
Correlation between transcriptomic data and qPCR results in SBPH. The relative expression level of each gene was determined by qPCR (blue) and was compared with the expression of the transcriptomic data (green). **a** UDP-glucuronosyltransferase, **b** venom serine carboxypeptidase-like, **c** sugar transporter, **d** maltase 2-like, **e** 60S ribosomal protein, **f** 40S ribosomal protein, **g** nucleotide exchange factor SIL1 I, **h** peroxisomal biogenesis factor 3-like, **i** 70 kDa heat shock protein, **j** peptide transporter family 2-like, **k** cytochrome P450, **l** ABC transporter of evm. TU.Contig86.54, **m** ABC transporter of evm. TU.Contig58.174

**Fig. 5:**
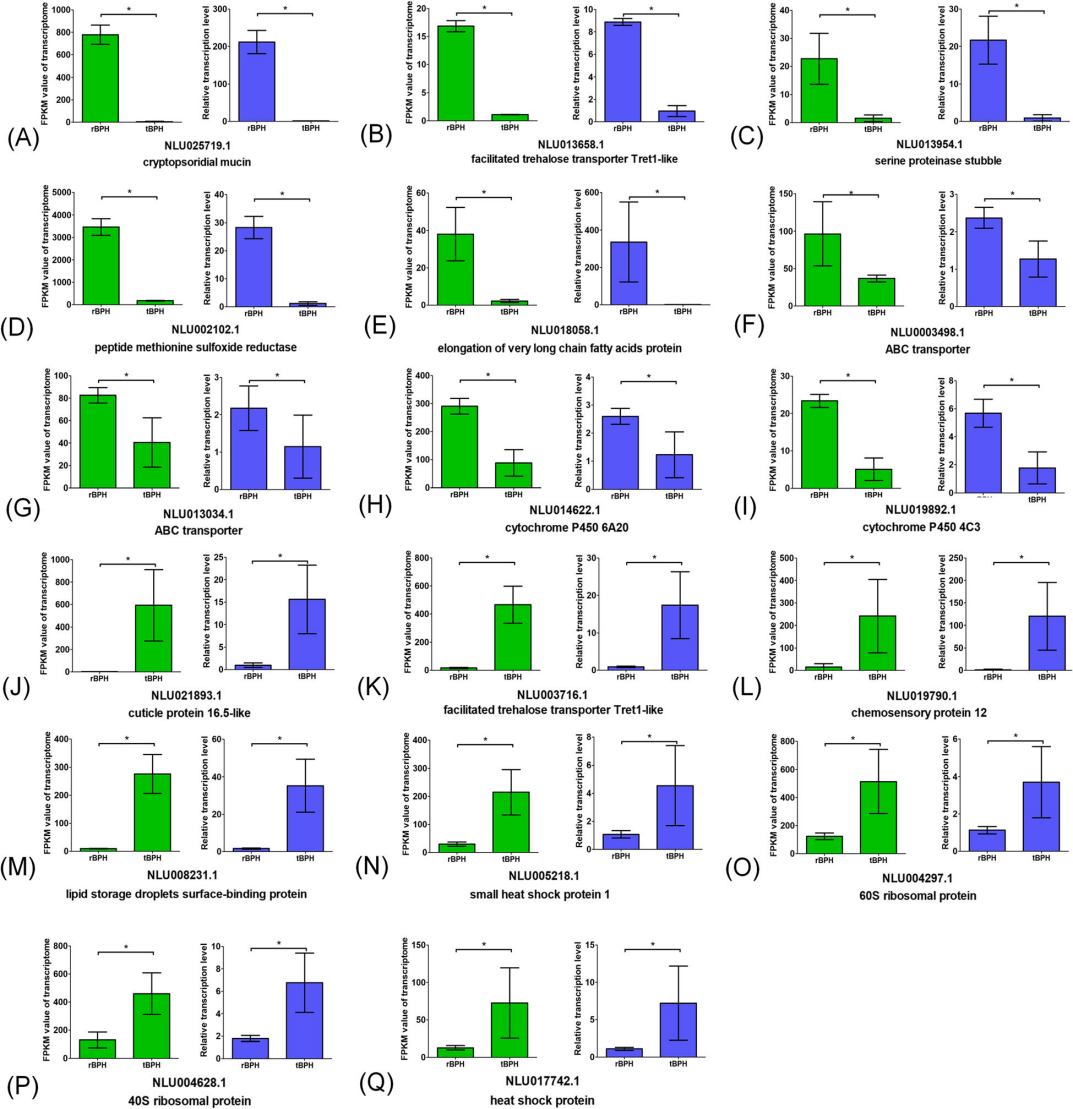
Correlation between transcriptomic data and qPCR results in BPH. The relative expression level of each gene was determined by qPCR (blue) and was compared with the expression of the transcriptomic data (green). **a** cryptopsoridial mucin, **b** facilitated trehalose transporter Tret1-like of NLU013658.1, **c** serine proteinase stubble, **d** peptide methionine sulfoxide reductase, **e** elongation of very long chain fatty acids protein, **f** ABC transporter of NLU003498.1, **g** ABC transporter of NLU013034.1, **h** cytochrome P450 6A20, **i** cytochrome P450 4C3, **j** cuticle protein 16.5-like I, **k** facilitated trehalose transporter Tret1-like of NLU003716.1, **l** chemosensory protein 12, **m** lipid storage droplets surface-binding protein, **n** small heat shock protein, **o** 60S ribosomal protein, **p** 40S ribosomal protein, **q** heat shock protein

To confirm the validity of the transcriptomic data, 15 SBPH genes and 18 BPH genes were selected for qPCR analysis. Thirteen SBPH genes (Fig. 4) and 17 BPH genes (Fig. 5) showed a concordant direction of change for the qPCR and transcriptomic results, indicating acceptable accuracy of the DEG transcriptomic results. The heat-shock proteins were significantly upregulated after BPH and SBPH transfer to wheat. The expression levels of the ABC transporters and cytochrome P450 were significantly increased after SBPH transfer to wheat but were significantly decreased in BPH under the same conditions. The ribosomal proteins were significantly downregulated in SBPH after transfer to wheat but were significantly upregulated in BPH after transfer. It is worth noting that two trehalose transporters in BPH showed significantly different changes in expression. The trehalose transporter NLU013658.1 was dramatically downregulated after BPH was transferred to wheat, but NLU003716.1 was dramatically upregulated under the same conditions. In SBPH, the nucleotide exchange factor, peroxisomal biogenesis factor, and peptide transporter were significantly upregulated after transfer, but the venom serine carboxypeptidase-like and maltase were significantly downregulated. In BPH, genes such as cryptosporidial mucin, serine proteinase stubble, and peptide methionine sulfoxide reductase were significantly downregulated after transfer, but the cuticle protein 16.5-like, chemosensory protein, and lipid storage droplets surface-binding protein were significantly upregulated.

## Discussion

The wSBPH and rSBPH used in this study were reared under laboratory conditions for more than 30 generations, whereas the tSBPH was collected by transferring rSBPH to wheat plants within 24 h. Surprisingly, the expression pattern of tSBPH was more similar to wSBPH than rSBPH (Fig. S2, Supporting information), indicating a rapid response by SBPH to the change in host plant and a leading role of the host plant in shaping gene expression. Additionally, the transfer of rSBPH to wheat plants gave rise to broad transcriptional readjustments, and the majority of DEGs were changed in the same direction in response to short-term transfer and long-term colonization. SBPH shifts between rice and wheat plants each year, whereas BPH seldom comes in contact with wheat plants [12, 13]. The frequent host transfer might help SBPH rapidly respond to new hosts, resulting in successful adaptation to wheat. A similar phenomenon was reported in *Tetranychus urticae*, which rapidly adapted to pre-exposed hosts [27]. In contrast, different expression patterns were revealed in 94.1% of homologous genes in BPH and SBPH when planthoppers were transferred to wheat plants (Table S5). Specifically, homologous genes associated with ribosomal proteins and detoxification were differentially regulated in the two species. With the majority of genes changed in the “wrong” direction, the low fitness of BPH on wheat plants might be determined within 24 h after transfer.

The genes involved in detoxification were significantly influenced when planthoppers were transferred to wheat plants (Fig. S3, Supporting information). In previous work, CYP4DE1 was found to be responsible for wheat adaptation, and a knockdown of CYP4DE1 significantly lowered the intestinal cell viability of SBPH reared on wheat plants [15]. In this study, we found that CYP4DE1 and other P450 genes were significantly upregulated after rSBPH was transferred to wheat plants, indicating that the induction of P450s might be critical. Similarly, 9 ABC transporters, which transport a large diversity of substrates across lipid membranes and out of cells, were also significantly upregulated in response to wheat plants. The involvement of ABC transporters in xenobiotic transport and insecticide resistance have been well documented [29]. These proteins have also been reported to participate in host adaptation in *Manduca sexta* [30], *Aphis nerii* [31], and *Chrysomela populi* [32]. The increased expression of ABC transporters signified that SBPH might accelerate the secretion of metabolites when feeding on wheat plants. Comparative analysis showed that the majority of differentially expressed detoxifying genes were upregulated when SBPH was transferred to wheat plants. However, these genes showed reduced expression in BPH (Fig. S3, Supporting information). We presumed that this might be associated with a resource-based metabolic trade-off [33]. When coping with such an adverse environment, it might become more critical for BPH to increase basic metabolism than to invest in the detoxification process. Further studies are needed to determine the variation in detoxification between BPH and SBPH on wheat plants.

Large numbers of ribosomal proteins were differentially expressed when the planthoppers were transferred to wheat plants. However, the majority of these genes showed different patterns of change between SBPH and BPH (Fig. S4, Supporting information). Most genes associated with standard roles in protein translation and ribosomal proteins are generally deemed to be stably expressed and are used as housekeeping genes [34]. However, recent studies have demonstrated that ribosomal proteins were differentially expressed in response to host transfers in *Bemisia tabaci* [35], *Helicoverpa armigera* [36], *Polygonia c-album* [37], and *Cryptolaemus montrouzieri* [38]. In this study, the expression level of ribosomal proteins was significantly downregulated when rSBPH was transferred to wheat but upregulated in rBPH under the same conditions. In an insect-plant model, regulation of ribosomal proteins is believed to counteract the ribosome inactivating proteins (RIPs), which are produced by host plants and inhibit protein synthesis in insects [39]. Different expression patterns of ribosomal proteins in the two planthoppers might influence the ability of the insect to counteract plant RIPs; this deserves further investigation. Additionally, ribosomal proteins play fundamental roles in the cellular process of translation [34]. The large-scale change of ribosomal proteins inevitably influences the regulation of other genes, which might partially explain the distinct variation in gene expression between the two planthopper species.

Genes associated with the amide/peptide biosynthetic process, amino sugar metabolic process, and aminoglycan metabolic process were significantly enriched in both SBPH and BPH after host transfer. Planthoppers are piercing-sucking herbivores that depend on phloem sap containing high concentrations of sugars, amino acids and inorganic ions as their food sources [15, 37]. Previous work has demonstrated that the sucrose concentration of rice phloem sap and wheat phloem sap is very similar, but the latter has a higher concentration of total amino acids compared with the former [19, 20]. In addition, the predominance of amides between the two phloem saps was also very different [19, 20]. In previous work, we found that the BPH genome lacks the ability to carry out de novo synthesis of some essential amino acids, requiring an additional supply from endosymbionts or plant phloem sap [18]. When planthoppers were sustained on different hosts, their endosymbiotic communities were changed [40]. Our presumption is that the difference in phloem sap composition or endosymbionts might influence nutritional status, which might result in a change in basic metabolism (particularly amino acid metabolic processes). This hypothesis has been partially verified by planthoppers reared on resistant rice, in which the physiological adaptation of insects to a novel rice variety was closely linked to a change in amino acid metabolism [41].

Sugar transporters were significantly differentially expressed when planthoppers were transferred to or colonized on wheat plants (Fig. S5, Supporting information). Sugar transporters mediate the movement of sugars into and out of cells in a diverse array of organisms and are vital for the utilization of ingested sugars as a nutritional resource and for the maintenance of osmotic balance in insects [42]. We found that the majority of differentially expressed sugar transporters belonged to facilitated trehalose transporters. Trehalose is the major hemolymph sugar in most insects [43]. Its concentration in hemolymph is determined by the balance between discharge from the fat body and uptake by other tissues via facilitated trehalose transporters [44]. In *Anopheles gambiae*, suppression of the trehalose transporter significantly diminished the hemolymph trehalose concentration, making the insect more sensitive to stress [45]. Similarly, the trehalose transporter from *Polypedilum vanderplanki* enhanced the desiccation tolerance of cell lines [46]. Involvement of the trehalose transporter in host adaptation has only been reported in a few insects [9]. However, increasing evidence demonstrates that trehalose, the regulatory product of the trehalose transporter, plays a key role in insects transitioning to different diets. For example, the trehalose concentration in hemolymph affected the food preference and carbohydrate intake in *Heliothis zea* and *Manduca sexta* [47, 48], and trehalose homeostasis was critical for *Drosophila* adapting to different dietary conditions [49]. In this study, the majority of sugar transporters were suppressed in SBPH and BPH reared on wheat plants. Accordingly, we inferred that the two planthoppers might use a similar strategy to maintain sugar homeostasis. However, it was also worth noting that two of the trehalose transporters in BPH were dramatically upregulated. BPH harbors many sugar transporter genes, and they differ in their function [50]. Further studies are needed to illustrate the function of sugar transporters in planthoppers coping with different diets.

Heat-shock proteins were prominently upregulated when the two planthopper species were transferred to or colonized wheat plants (Fig. S5, Supporting information). Heat-shock proteins are well-known stress proteins that respond to an array of stresses including thermal hardening, oxidative hardening, chemical pesticides, and desiccation [51, 52]. The induced expression of these proteins as a significant molecular chaperone prevents the irreversible denaturation of proteins and enhances the coping capacity of insects in the face of stress [51]. Previously, the influence of diet quality on heat-shock proteins expression was described in *Drosophila melanogaster*, and diet-induced heat-shock proteins increased the heat and desiccation tolerance of flies [53]. Feeding on wheat was a biotic stressor for BPH and SBPH [16], which might result in increased heat-shock protein expression.

## Conclusions

Overall, SBPH successfully coped with wheat hosts, and the majority of DEGs responded similarly to short-term transfer and long-term colonization. Compared with SBPH, BPH showed distinct gene expression changes after transfer to the wheat plants. The different pattern of changes in gene expression between BPH and SBPH might account for their adaptive differences on wheat plants. Specifically, genes associated with sugar transporters and heat-shock proteins showed similar expression trends for BPH and SBPH. Other genes associated with detoxification, ribosomal proteins, and amino acid metabolism were regulated differently between the two planthoppers. Our work increases our knowledge of planthoppers adapting to rice and wheat hosts, and might be useful in pest management.

## Methods

### Insect strains

The SBPH and BPH populations used in this study were originally collected from a rice field at Huajiachi Campus, Zhejiang University, Hangzhou, China. This rice field (30.271 ° N, 120.199 ° E) was specifically used for field experiments. The rSBPH and wSBPH were generated by rearing SBPHs on fresh rice (Xiushui 134) and wheat (Luyuan 502) plants for over 30 generations. The rBPH have been reared on rice (Xiushui 134) plants for more than 40 generations. All insects were reared in large cages, and more than 300 individuals were used for propagation in each generation.

### Survival analysis

Survival analysis of rSBPH on different hosts has been done previously [28]. However, the performance of wSBPH, as well as rBPH, on wheat plants remained unknown. For BPH, 3rd instar nymphs (rBPH) were reared on rice plants (4–5-leaf stage) or were transferred to wheat plants (4–5-leaf stage). The rBPH that provided with water only were used as a control. For SBPH, rSBPH were reared on rice plants (4–5-leaf stage) or were transferred to wheat plants (4–5-leaf stage), and wSBPH were reared on wheat plants (4–5-leaf stage). We recorded the survival rates of each treatment every day. Three biological replicates were conducted for each treatment. Each replicate contains a group of 40–50 insects (approximately 4–5 insects per plant). The log-rank test in SPSS 19.0 (SPSS, Chicago, IL, USA) was used to determine the statistical significance of survival distributions for each treatment group.

### Transcriptomic sequencing

For rSBPH, wSBPH, and rBPH populations, newly emerged 5th instar nymphs were collected and continually reared on their original hosts for 24 h. For tSBPH and tBPH populations, newly emerged 5th instar nymphs (maintained on rice plants) were collected and transferred to wheat plants for 24 h. The nymphs were anesthetized on ice and their guts were carefully dissected under a stereomicroscope (COIC, Chongqing, China). Total RNA extraction was performed using TRIzol Total RNA Isolation Kit (Takara, Dalian, China) according to the manufacturer’s instructions. Three replicates (all replicates were collected from the same insect strain) were performed for each treatment, and each replicate contained a pool of 100 guts.

The RNA samples were sent to Beijing Genomics Institute (BGI, Beijing, China) for transcriptomic sequencing as we previously described [54]. A BGISEQ-500 platform (BGI, Beijing, China) was used for library sequencing. After filtering the low quality reads using the internal software, the clean reads from each cDNA library were aligned to reference genome sequences of *Nilaparvata lugens* (BioProjects: PRJNA398259; Link: https://www.ncbi.nlm.nih.gov/genome/2941?genome_assembly_id=209423) [18] and *Laodelphax striatellus* (BioProjects: PRJNA393384; Link: https://www.ncbi.nlm.nih.gov/genome/12198?genome_assembly_id=449717) [21] using Hierarchical Indexing for Spliced Alignment of Transcripts (HISAT). The clean data of rSBPH, rBPH, wSBPH, tSBPH, and tBPH have been submitted to the database of the NCBI Sequence Read Archive under the accession number PRJNA564687.

The DEGseq software [55, 56] was used to analyze the DEGs, and genes with log2-ratio > 1 and adjusted *p* value < 0.05 were identified. Kyoto Encyclopedia of Genes and Genomes (KEGG) enrichment analysis and Gene Ontology (GO) were based on the KEGG pathway database (http://www.genome.jp/kegg/) and GO Database (http://www.geneontology.org/).

### Gene family identification

Previous work demonstrated that genes associated with detoxification, digestion, and transport played critical roles in host transfer [7]. To analyze these genes in BPH and SBPH, the target genes were firstly identified according to their gene annotations. Then, the identified genes were used as queries to BLAST against the genomic databases of *Nilaparvata lugens* [18] and *Laodelphax striatellus* [21] using local BLAST (ftp://ftp.ncbi.nlm.nih.gov/blast). Finally, we used the non-redundant (nr) protein database in National Center for Biotechnology Information (NCBI) to validate the gene annotation.

### Comparative genomic analysis

The protein families of BPH and SBPH were analyzed using the ORTHOMCL software 2.0.3 (http://orthomcl.org/common/downloads/software/v2.0/) according to the method we previously described [57]. Briefly, the deduced amino acid sequences of BPH and SBPH were firstly downloaded from NCBI (BioProjects: PRJNA398259 and PRJNA393384). Then, all-vs-all BLASTp algorithm was used to find the homologous pairs of sequences with an E-value of <1e-5. The BLASTp result was subsequently converted into a normalized similarity matrix. The matrix was then analyzed by a Markov chain clustering (MCL) for clustering [58]. We use an inflation factor of 1.5 to control the cluster tightness.

### qPCR analysis

qPCR analysis was used to confirm the validity of transcriptomic data. Sample collection and RNA extraction was performed as described above. The concentration of each RNA sample was determined by NanoDrop ND-1000 spectrophotometer (NanoDrop Technologies, Wilmington, DE, USA). Reverse transcription was performed by HiScript II Q RT SuperMix (Vazyme, Nanjing, China), and 1 μg RNA was used in a 20-μl reaction system. RNA with no-reverse-transcriptase was used as a negative control. The primers used for qPCR were designed by Primer Premier 6.0 and listed in Table S6. We use two housekeeping genes (β-actin and GAPDH) as internal controls. qPCR was performed using the ABI 7500 Real-Time PCR System (Applied Biosystems, Carlsbad, CA) and the SYBR Green Supermix Kit (Yeasen, Shanghai, China). The program was run under the following conditions: denaturation at 95 °C for 5 min, followed by 40 cycles at 95 °C for 10 s and 60 °C for 30 s. qPCR result was calculated according to a relative quantitative method (2^-∆∆Ct^) [59]. Three independent biological replicates were performed.

## Supplementary information

**Additional file 1: Figure S1.** Saturation analysis of 15 sequencing libraries.

**Additional file 2: Figure S2.** Principal component analysis (PCA) of gene expression patterns in SBPH **(A**) and BPH **(B).** The first two principal components (PC1 and PC2) based on transcriptomic results are shown with each plot representing one sample.

**Additional file 3: Figure S3.** The expression pattern of detoxification-related genes. The differentially expressed genes associated with ABC transporters, cytochrome P450s, UDP-glucuronosyltransferases (UGTs), and esterases in SBPH (A) and BPH (B) are illustrated in the heat map.

**Additional file 4: Figure S4.** The expression pattern of ribosomal proteins. The differentially expressed genes associated with ribosomal proteins in SBPH (A) and BPH (B) are illustrated in the heat map.

**Additional file 5: Figure S5.** The expression pattern of sugar transporters and heat-shock proteins. The differentially expressed genes associated with trehalose transporters, sugar transporters, and heat-shock proteins in SBPH (A, C) and BPH (B, D) are illustrated in the heat map.

**Additional file 6: Table S1.** Differentially expressed genes when rice-colonized SBPH (rSBPH) were transferred to wheat plants.

**Additional file 7: Table S2.** Differentially expressed genes when rice-colonized BPH (rBPH) were transferred to wheat plants.

**Additional file 8: Table S3.** Differentially expressed genes in rice-colonized SBPH (rSBPH) and wheat-colonized SBPH (wSBPH).

**Additional file 9: Table S4.** Classification of the identified differentially expressed genes in SBPH.

**Additional file 10: Table S5.** Comparative genomic analysis of genes in response to host transfer in two planthopper species.

**Additional file 11: Table S6.** Primers used for qPCR.

## Data Availability

All sequencing data generated in this study were submitted to the NCBI Sequence Read Archive under accession number PRJNA564687. Other related data are available within the manuscript and its additional files. The genome data of *Nilaparvata lugens* [18] and *Laodelphax striatellus* [21] were downloaded from NCBI under the BioProject: PRJNA398259 and PRJNA393384.

## Abbreviations

BPH: Brown planthopper
DEG: Differentially expressed gene
GST: Glutathione S-transferase
MCL: Markov chain clustering
PCA: Principal component analysis
RIP: Ribosome inactivating protein
rBPH: Brown planthopper that colony on rice
rSBPH: Small brown planthopper that colony on rice
SBPH: Small brown planthopper
tBPH: Brown planthopper that transferred from rice to wheat
tSBPH: Small brown planthopper that transferred from rice to wheat
UGT: UDP-glucosyltransferase
wSBPH: Small brown planthopper that colonized on wheat
