# Supplementary figures and images for: Comparative analysis of diet-associated responses in two rice planthopper species
Source: BMC Genomics. 2020 Aug 17;21:565. doi: 10.1186/s12864-020-06976-2 (PMC7437935; doi:10.1186/s12864-020-06976-2)

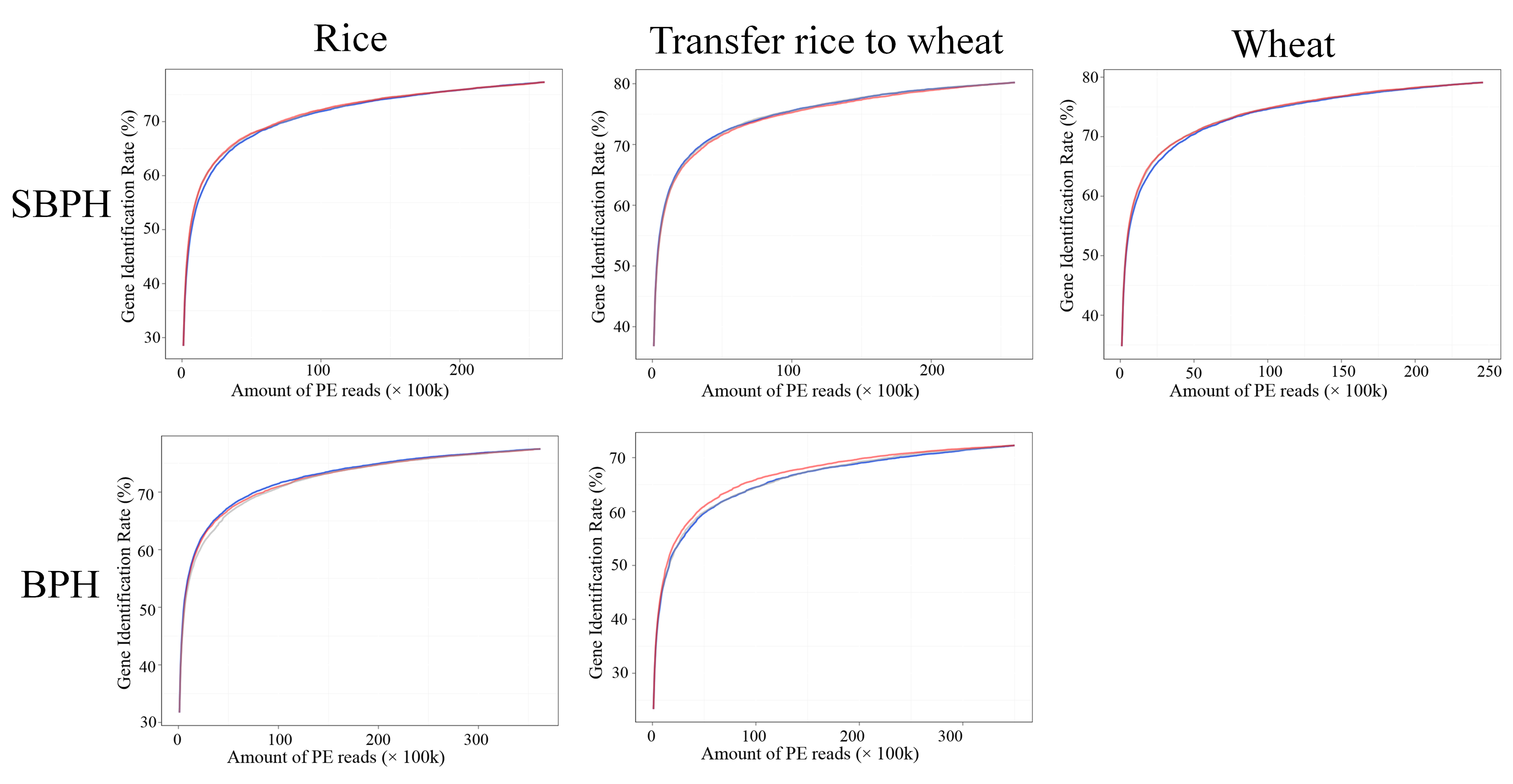

Supplement: Supplementary file 1 — Additional file 1: Figure S1. Saturation analysis of 15 sequencing libraries. [file 12864_2020_6976_MOESM1_ESM.tif]

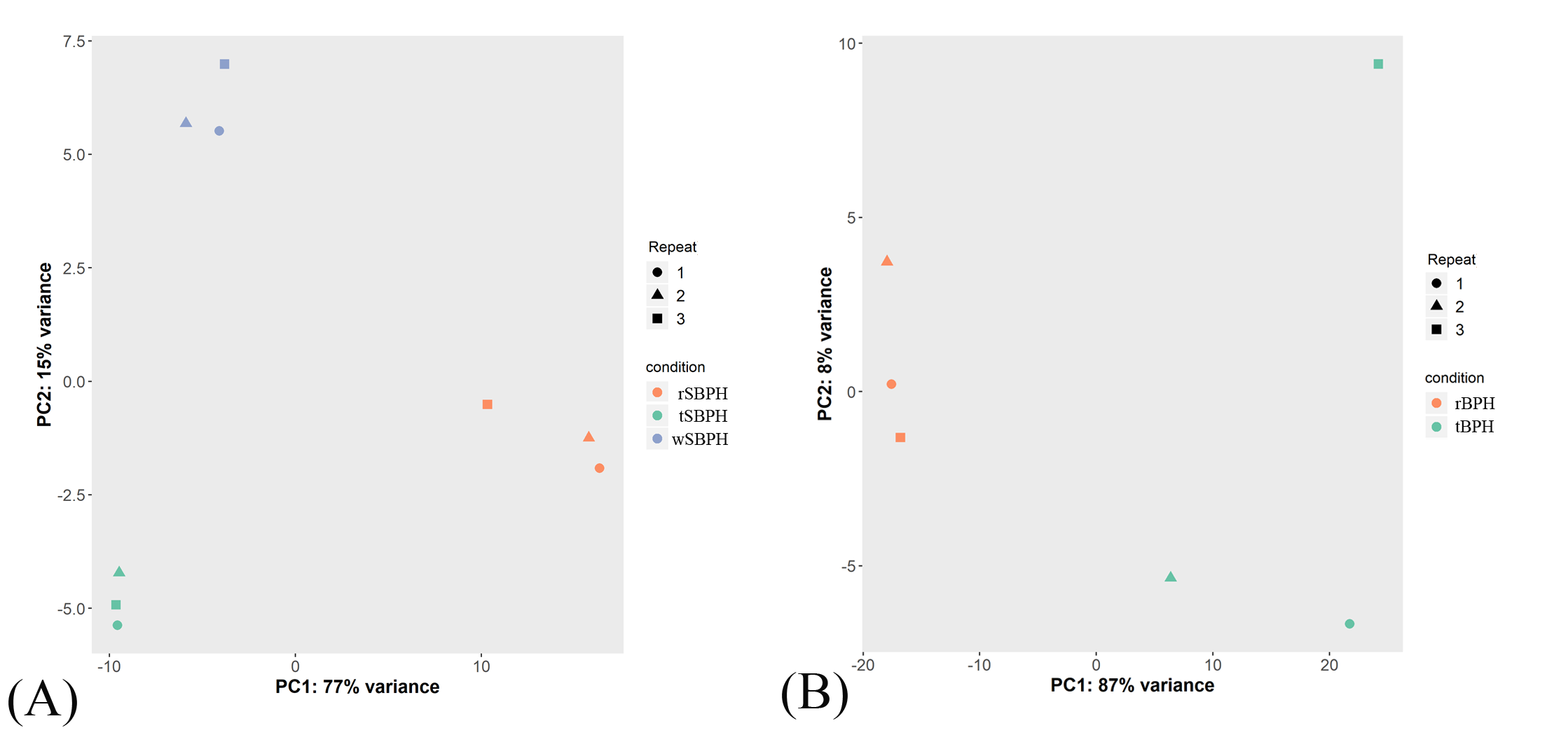

Supplement: Supplementary file 2 — Additional file 2: Figure S2. Principal component analysis (PCA) of gene expression patterns in SBPH (A) and BPH (B). The first two principal components (PC1 and PC2) based on transcriptomic results are shown with each plot representing one sample. [file 12864_2020_6976_MOESM2_ESM.tif]

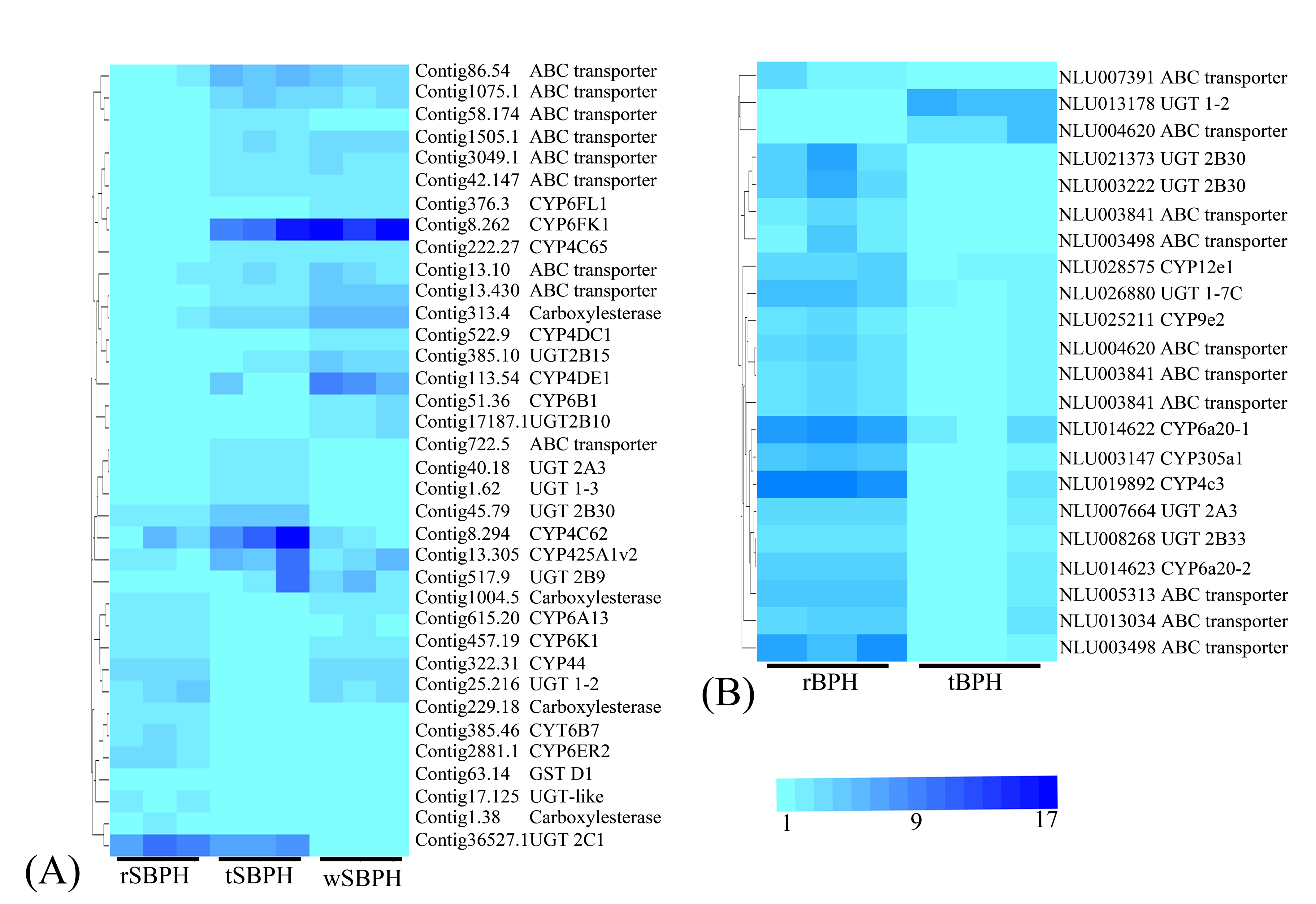

Supplement: Supplementary file 3 — Additional file 3: Figure S3. The expression pattern of detoxification-related genes. The differentially expressed genes associated with ABC transporters, cytochrome P450s, UDP-glucuronosyltransferases (UGTs), and esterases in SBPH (A) and BPH (B) are illustrated in the heat map. [file 12864_2020_6976_MOESM3_ESM.tif]

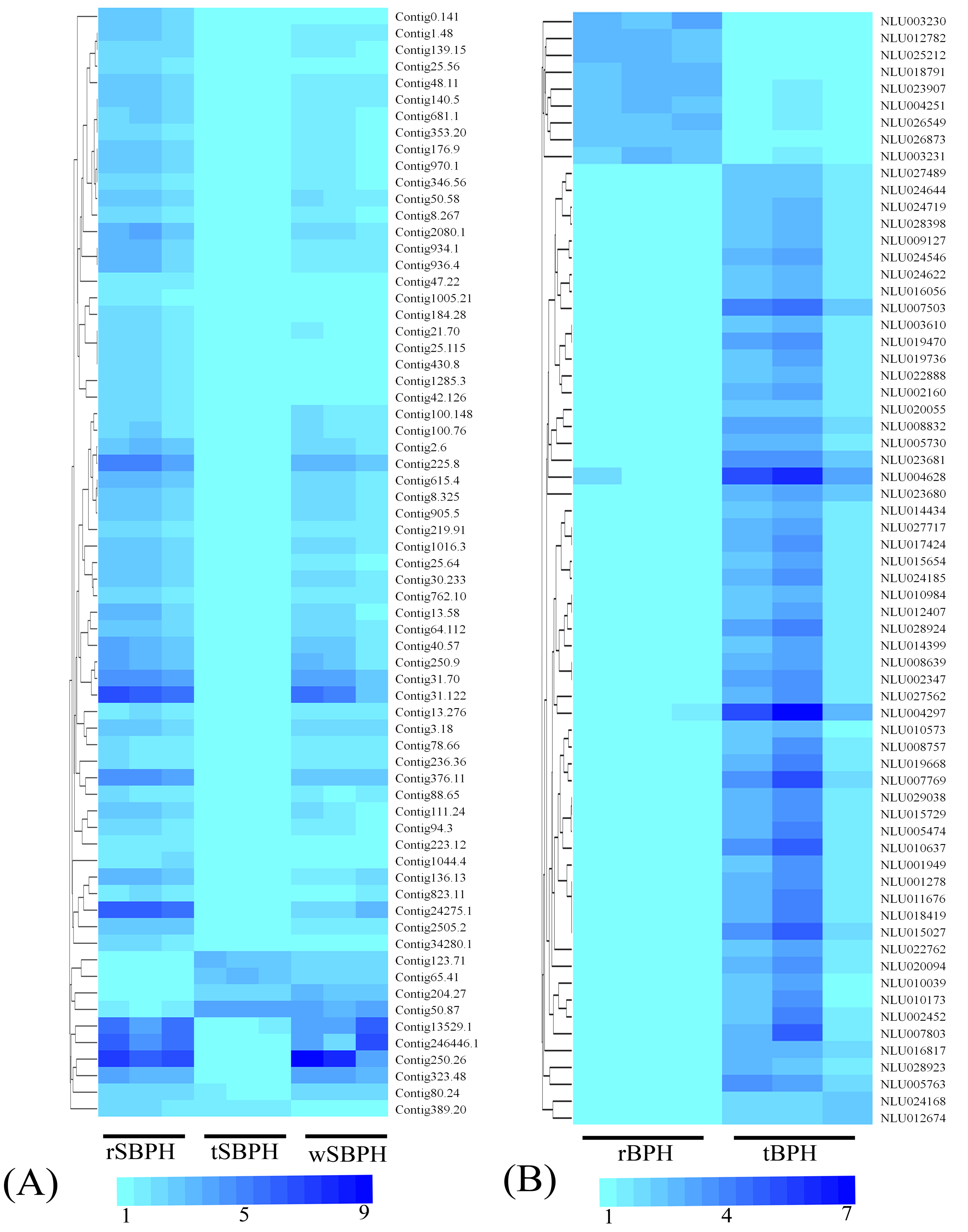

Supplement: Supplementary file 4 — Additional file 4: Figure S4. The expression pattern of ribosomal proteins. The differentially expressed genes associated with ribosomal proteins in SBPH (A) and BPH (B) are illustrated in the heat map. [file 12864_2020_6976_MOESM4_ESM.tif]

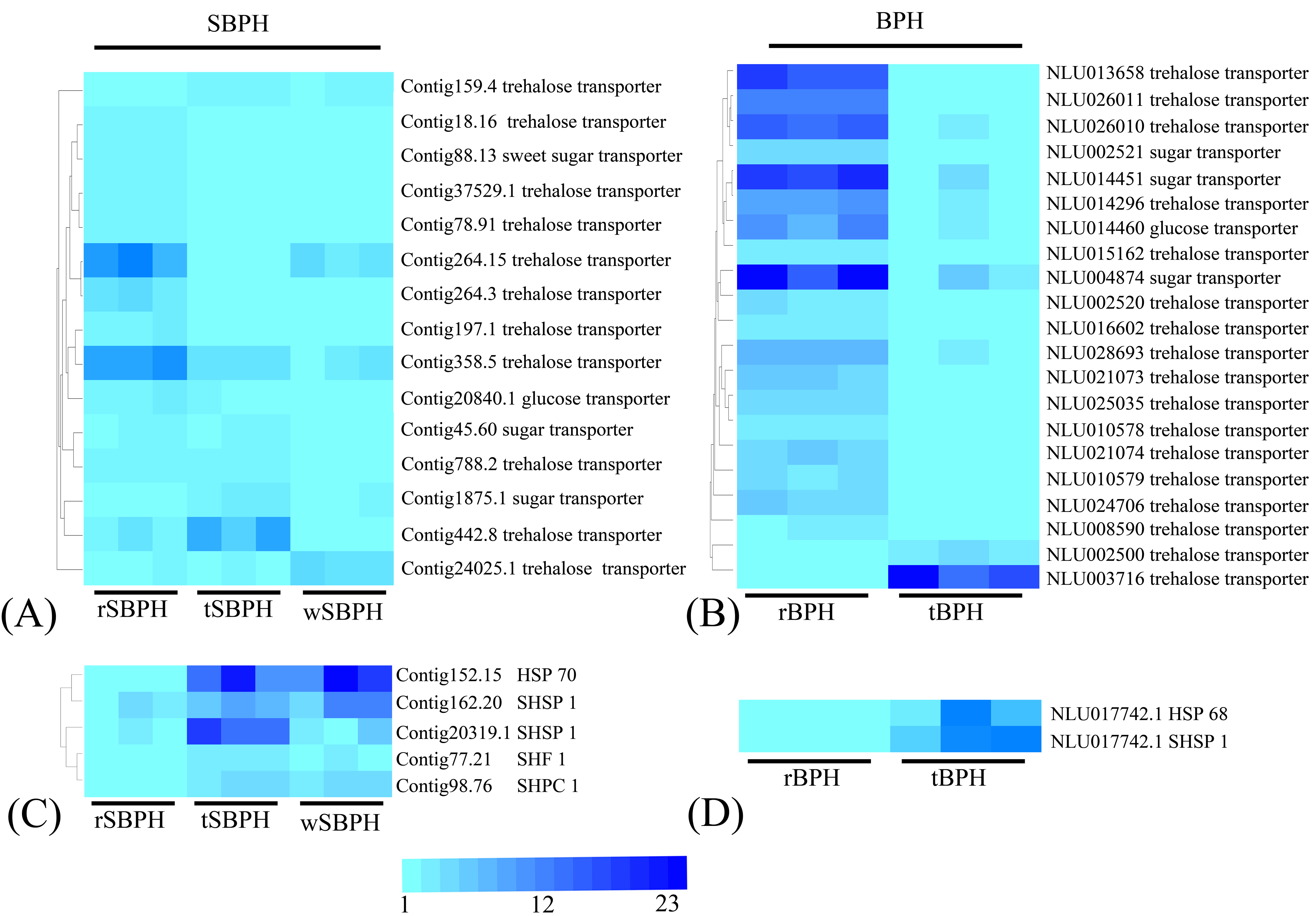

Supplement: Supplementary file 5 — Additional file 5: Figure S5. The expression pattern of sugar transporters and heat-shock proteins. The differentially expressed genes associated with trehalose transporters, sugar transporters, and heat-shock proteins in SBPH (A, C) and BPH (B, D) are illustrated in the heat map. [file 12864_2020_6976_MOESM5_ESM.tif]
