# Supplementary material for: Comparative analysis of diet-associated responses in two rice planthopper species
Source: BMC Genomics. 2020 Aug 17;21:565. doi: 10.1186/s12864-020-06976-2 (PMC7437935; doi:10.1186/s12864-020-06976-2)
Supplement: Supplementary file 11 — Additional file 11: Table S6. Primers used for qPCR. [file 12864_2020_6976_MOESM11_ESM.doc]

**Table S6 Primers used for qPCR**

| **Genes** | **Forward primer (5’-3’)** | Reverse primer (5’-3’) |
| --- | --- | --- |
| *LsGAPDH* | GTGTTGACTACATGGTCTACT | GCTCACTGAATACCTGGATT |
| *LsActin* | AATCGTAAGAGACATCAAGGAG | AGGCAATTCGTAGGACTTCT |
| *evm.TU.Contig36527.1* | CATTGGTTGACGGACATTATG | CTGAGGAAGCGTATTGTTCTT |
| *evm.TU.Contig358.5* | GTGGCATAGCGTTATCTTACT | TACAGGTAGACAGGTGATTCA |
| *evm.TU.Contig1928.1* | CATAACATGACATCCGAACTG | GTAGGACATCGTATGCTCTATA |
| *evm.TU.Contig191.3* | GTAAGAGACGATGGTAGGAATA | AAGTTCTCAGTCAACGGATAG |
| *evm.TU.Contig8.262* | GTGCTTCTTAATCTGCCATAC | GAATGAACTTCTCTGGATTGG |
| *evm.TU.Contig152.15* | CACTGAGAAGATTGAGAACTG | GTAGTGTGGAACGGAATAGAT |
| *evm.TU.Contig850.14* | CCTATCTCAAGCAAGAACAAC | CCACATAACAGCAGAATAGTAG |
| *evm.TU.Contig25.5* | CGCTGTCTTACCTTATAGTTG | GATTCTCCACTCAATTCCTTC |
| *evm.TU.Contig11687.1* | GCTTATGCTTGAACGATCTAG | TCATCATGCTGGTGTATCTTC |
| *evm.TU.Contig36527.1* | CATTGGTTGACGGACATTATG | CTGAGGAAGCGTATTGTTCTT |
| *evm.TU.Contig358.5* | GTGGCATAGCGTTATCTTACT | TACAGGTAGACAGGTGATTCA |
| *evm.TU.Contig1928.1* | CATAACATGACATCCGAACTG | GTAGGACATCGTATGCTCTATA |
| *evm.TU.Contig86.54* | GAGGAGGAATATGAGGTAGAC | GTATCTGGCTCTTGATTCTTC |
| *evm.TU.Contig58.174* | CAATTCTAGCTGTGCATACCT | TAGCCGAGTTCTGAATGATAG |
| *evm.TU.Contig31.122* | CGTTCTTCGTTGAAGTGATTG | CAGAGGTTCAAACTGTATTTCC |
| *evm.TU.Contig225.8* | GACGAAGAAGGATAAGAAGAAC | CTCATCTGGGTTAGAATGTTAC |
| *NlGAPDH* | CACTCAGTAACATCTTCTCAG | CCTTCAAGGCTTGGTATAAC |
| *NlActin* | GTGCGTGACATCAAGGAGAAGC | GGAAGGAAGGCTGGAACAGAG |
| *NLU025719.1* | CCTTTCTACTACCCTTACAACA | GCTCTTCTCACTGCTATATGG |
| *NLU013658.1* | CAATGTATTTGGCGGAGATAG | ATAGTAAGGCGACTCAGGAAT |
| *NLU013954.1* | ATTGTGTCCTACTTGTCCTTC | TACTACTAATCCTCCTGCTACT |
| *NLU002102.1* | GAAGCCATTGACATTGACTAC | GGAATGACTTTAGTGACGATG |
| *NLU018058.1* | ACTGCTCTACACCTTACTCTA | CTGTACCGTAGTCTACTTGAA |
| *NLU021893.1* | GAGGAGAAGAGGGACAAGAAG | CAGCAGAGTAGGAGGAAACAG |
| *NLU003716.1* | GAGAATACCACGATGAAGAGA | GGCTATATGTCTGATTGTCTTG |
| *NLU019790.1* | AACTACTACAAGTGTCTGCTG | GTATTCGGTTGGCTTCTTCTC |
| *NLU008231.1* | TTCTAAGTACAGCCGAGAGTA | CATAATCCAATCCTGAACAGAG |
| *NLU003498.1* | TAGTCAGTCAGGAACCAGTAT | GTGATGAAGTCGTAAGCATTG |
| *NLU013034.1* | GCTAATGTATGCTATCCTGATG | GTCCAGTGATATACTCGTAGAT |
| *NLU014622.1* | GTGACCGTAATCTACTACTTC | CTGTTGTATGAGTTCAGGATC |
| *NLU019892.1* | GACTGGAGGAAACCTTACATA | GCAGTTTAGGTCCATATTTCC |
| *NLU004297.1* | CAGAAGCTTGCCAAGAAATTG | TTGGTTCTCCTCCAGTGTCTA |
| *NLU004628.1* | CAAGAACGGAAAGAAGATCAC | CTTAACCACCTTGAACCTGAC |
| *NLU017742.1* | GATATTGACAGGATGGTGAATG | CACACTGAAGATGTAGTTCTC |
| *NLU005218.1* | TCTCTGCTACCGATTATCCTT | ATCATCGTTCAGCAATCCAAG |
